# Composition of Maternal Circulating Short-Chain Fatty Acids in Gestational Diabetes Mellitus and Their Associations with Placental Metabolism

**DOI:** 10.3390/nu14183727

**Published:** 2022-09-09

**Authors:** Shuxian Wang, Yu Liu, Shengtang Qin, Huixia Yang

**Affiliations:** 1 Department of Obstetrics and Gynaecology, Peking University First Hospital, Beijing 100034, China; 2 Beijing Key Laboratory of Maternal Fetal Medicine of Gestational Diabetes Mellitus, Beijing 100034, China

**Keywords:** short-chain fatty acid (SCFAs), immune, metabolism, placental metabolome, gestational diabetes mellitus (GDM)

## Abstract

Short-chain fatty acids (SCFAs), which are produced by gut microbiota from dietary fiber, have become candidates for gestational diabetes mellitus (GDM) treatment. However, the associations of circulating SCFAs with maternal–neonatal clinical parameters in GDM and further influences on placental immune–metabolic responses are unclear. Acetate, propionate, and butyrate were decreased in GDM during the second and third trimesters, especially in those with abnormal glucose tolerance at three “oral glucose tolerance test” time points. Butyrate was closely associated with acetate and propionate in correlation and dynamic trajectory analysis. Moreover, butyrate was negatively correlated with white blood cell counts, neutrophil counts, prepregnancy BMI, gestational weight gain per week before GDM diagnosis, and ponderal index but positively correlated with total cholesterol and low-density lipoprotein levels in all pregnancies. On the premise of reduced SCFA contents in GDM, the placental G-protein-coupled receptors 41 and 43 (GPR41/43) were decreased, and histone deacetylases (HDACs) were increased, accompanied by enhanced inflammatory responses. The metabolic status was disturbed, as evidenced by activated glycolysis in GDM. Maternal circulating acetate, propionate, and butyrate levels were associated with demographic factors in normal and GDM women. They influenced placental function and fetal development at birth through GPRs or HDACs, providing more evidence of their therapeutic capacity for GDM pregnancies.

## 1. Introduction

Gestational diabetes mellitus (GDM) refers to diabetes diagnosed in the second or third trimester of pregnancy in the absence of overt diabetes diagnosed prior to gestation [1]. GDM is one of the most common pregnancy complications and has been associated with preeclampsia, fetal macrosomia, and long-term adverse effects, such as type 2 diabetes (T2DM) and cardiovascular diseases [2]. In addition, children born to mothers with GDM are at increased risks of obesity, metabolic syndrome, and T2DM later in life [3]. Therefore, timely intervention is critical for both mothers and their offspring. Emerging evidence has shown the influences of the gut microbiome on GDM development. Short-chain fatty acid (SCFA)-producing genera, including *Faecalibacterium*, *Ruminococcus*, *Bifidobacterium*, and *Akkermansia* were found to be reduced in individuals with GDM [4,5]. These results indicate that some of this dysbiosis is mediated by SCFAs.

SCFAs, examples of which include acetic acid, propionic acid, and butyric acid, are fermentation products resulting from dietary fiber produced by gut bacteria and interact with the host metabolism as energetic substrates or signaling molecules [6]. SCFAs are absorbed in the intestine, and most butyric acids are utilized by colonocytes, whereas acetic and propionic acids are metabolized and involved in gluconeogenesis and lipogenesis [7]. Gut microbiota modification (such as by SCFA-producing probiotics) or SCFA supplementation are potential treatments for GDM. Insulin resistance was ameliorated and lipid metabolism was improved in a GDM group treated with a combination capsule of *Lactobacillus acidophilus*, *Lactobacillus casei*, and *Bifidobacterium bifidum* for six weeks during the middle trimester [8]. Oral butyric acid supplementation was found to improve insulin sensitivity and to increase energy expenditure in mice [9]. In obese pregnant women, serum propionate exerted protective effects and was negatively correlated with maternal leptin levels, newborn length, and body weight [10]. As signaling products, SCFAs can inhibit histone deacetylase (HDAC) activity to promote gene transcription or activate G-protein-coupled receptors (GPRs), especially GPR43 and GPR41, for involvement in immune–metabolic responses [11,12]. GPRs are ubiquitously expressed in placental trophoblast, amnion, adipose tissue, etc. Elevated GPR41 and GPR43 expression was observed in gestational tissue during parturition and was found to exert functional effects by dampening proinflammatory cytokine responses and inhibiting the neutrophil chemotaxis [13]. In addition, SCFA pathways are compromised in GDM pregnancies, as indicated by reduced acetate, butanoate, and propanoate levels during late gestation [14]. SCFA addition inhibited inflammation and oxidative stress by preventing extracellular regulated protein kinase (ERK) activation in an in vitro GDM model established via lipopolysaccharide (LPS) and tumor necrosis factor-α (TNF-α) stimulation [15].

GDM is characterized by enhanced peripheral insulin resistance (IR) and low-grade inflammation. The release of placenta-derived TNF-α and leptin is initiated and exacerbated by insulin receptor substrate 1 (IRS-1) serine phosphorylation [16,17]. Most fetal growth occurs in the second and third trimesters. Reduced SCFA contents in circulation may be involved in this pathological process by interacting with the placenta, which may further influence fetal development. In addition, dysregulations in carbohydrate metabolism, such as increased glycolysis and decreased fatty acid oxidation, are hallmarks of GDM [18]. Studies have shown that glucose transporter 1 (GLUT1) expression is increased in GDM placentae and that glycolytic genes are upregulated in adipose tissue [19,20,21]. However, the therapeutic capacity of circulating SCFAs and their associations with systemic and placental abnormalities complicated by GDM are unclear. Combined with the antidiabetic and anti-inflammatory effects of SCFAs, we aimed to provide more evidence of the therapeutic potential of SCFAs by (1) comparing SCFA contents between GDM and normal pregnancies in the second (T2) and third (T3) trimesters, (2) investigating the associations of SCFAs with maternal–fetal clinical characteristics, and (3) investigating their roles in placental immunometabolism processes.

## 2. Materials and Methods

### 2.1. Study Participants

Participants were enrolled at Peking University First Hospital between October 2017 and July 2019. Eligible participants had a singleton fetus and were younger than 35 years old. The major exclusion criteria included a history of type 1 or 2 diabetes, gastrointestinal diseases, preeclampsia, hypertension disorders, smoking or alcohol consumption habits, and long-term medicine or prebiotic use.

GDM was diagnosed using the International Association of Diabetes and Pregnancy Study Groups (IADPSG) criteria and a 75 g oral glucose tolerance test (OGTT) between 24 and 28 weeks of gestation [1]. Accordingly, four pregnancy subgroups were generated: (1) normal; (2) GDM subtype 1: isolated elevated glucose level under fasting; (3) GDM subtype 2: elevated glucose levels at 1 h and/or 2 h with normal fasting glucose level; and (4) GDM subtype 3: elevated glucose levels under fasting and at either 1 h or 2 h [22]. All pregnant women diagnosed in our hospital were placed on lifestyle treatment and self-glucose monitoring. In total, 40 normal and 34 GDM pregnancies were selected.

This project was approved by the Ethics Committee of Peking University First Hospital (V2.0/201504.20), and informed consent was obtained from all participants.

### 2.2. Sample Collection

The samples were collected by well-trained staff according to standard operating procedures. Maternal fasting blood was drawn into prechilled EDTA tubes during each trimester and centrifuged at 2000 rpm for 20 min to prepare plasma. The placentae were obtained at term within 30 min after delivery, and fragments of villous tissue were isolated from the basal plate at sites located 5 cm from the umbilical cord insertion site. The plasma and tissues were aliquoted and stored at −80 ℃ until laboratory analysis.

### 2.3. Anthropometrics and Biochemical Assessment

Clinical information, such as demographic factors (maternal age; prepregnancy weight and height; last menstrual period (LMP); gestational age; delivery mode; neonatal birth weight, height, and head circumstance; and placental weight and volume) were collected from medical records. In particular, gestational age was determined by self-reported LMP and corrected by ultrasound dating. Body weight was measured multiple times during pregnancy until childbirth, and total gestational weight gain (GWG) or GWG per week was calculated according to the latest standard.

Prepregnancy BMI (*p*-BMI) was calculated as weight divided by squared height (kg/m^2^). The fetus–placenta ratio (FPR) was calculated by dividing placental weight by birth weight. The ponderal index (PI) was evaluated as 100 times the birth weight divided by the cube of birth length (g/cm^3^).

Other clinical information, such as white blood cell count (WBC), neutrophil (NEU) count, and OGTT results, was obtained from medical records, and the area under the curve (AUC) for glucose was calculated following the trapezoidal rule. Fasting glucose levels, insulin levels, and lipid profiles, including total cholesterol (TCHO), total triglycerides (TGs), high-density lipoprotein levels (HDL), and low-density lipoprotein (LDL) levels, were obtained in our hospital.

### 2.4. Measurement of SCFAs and Energy Metabolism Intermediates by LC-MS/MS

SCFA contents were measured in plasma, and substrates for energy metabolism were detected in the placenta. Briefly, 100 µL plasma and 100 mg placenta tissues were thawed at 4 °C and mixed with 400 μL or 1 mL cold methanol/acetonitrile (1:1, *v*/*v*) to remove the protein. The placental samples were sonicated at a low temperature (30 min/once, twice) before centrifugation (20 min, 14,000× *g*, 4 °C). Then, the supernatants were dried in a vacuum centrifuge and resolved in 100 μL acetonitrile/water (1:1, *v*/*v*) for LC-MS/MS analysis. Analyses were performed using a UHPLC (1290 Infinity LC, Agilent Technologies, Palo Alto, CA, USA) coupled with a QTRAP instrument (AB Sciex 5500) using an ACQUITY UPLC BEH amide column (2.1 × 100 mm, 1.7 μm, Waters MS Technologies, Manchester, UK). MS/MS analysis (MRM) was performed in ESI negative mode. Data acquisition and processing were accomplished using Multiquant software (AB SCIEX, Boston, MA, USA).

### 2.5. RNA Isolation and Reverse-Transcription Polymerase Chain Reaction (RT–PCR)

Total RNA from placental tissues was extracted using TRIzol reagent (Invitrogen, Carlsbad, CA, USA) according to the manufacturer’s instructions, and cDNA was synthesized from 2 µg of RNA using a FastKing RT kit with DNase (Tiangen Biotech, Beijing, China). Gene expression analysis was evaluated by RT–PCR using an ABI Power SYBR Green gene expression system (Applied Biosystems, Waltham, MA, USA) on an ABI 7500 sequence detection system. The relative expression levels of mRNA were normalized to 18S RNA, and the fold changes were calculated using the 2-^(ΔΔCt)^ method. The primer sequences used in the experiments are described in Supplementary Table S1.

### 2.6. Statistical Analysis

The results are expressed as the mean ± standard deviation (SD) or medians (quantiles) for continuous variables. Comparisons were performed by Student’s t test between two groups or one-way analysis of variance (ANOVA), followed by a post hoc test among more groups for normally distributed variables. A Mann–Whitney U test and Kruskal–Wallis test were performed for variables with skewed distributions. The area under the receiver operating characteristic (ROC) curve was calculated, setting the OGTT results as the gold standard. Pearson’s coefficient was adopted for correlation analysis between SCFAs, SCFAs, and demographic characteristics or metabolic blood measurements. The correlation coefficients of repeated observations were calculated using the methods described in previous studies [23,24]. We applied a multivariable linear regression model to evaluate the association between SCFA levels and maternal and/or neonatal characteristics, adjusting for confounding factors, such as *p*-BMI. A logistic regression model was used to examine the key factors with respect to GDM occurrence. SPSS 26.0 statistical software (SPSS Inc., Chicago, IL, USA) was used for statistical analysis. Heatmaps were generated using the ggplot2 R package, and other analyses were conducted by GraphPad Prism 8.0 (GraphPad Co. Ltd., San Diego, CA, USA). A *p* value < 0.05 was considered statistically significant. Statistically significant differences are shown as follows: **** *p* < 0.0001, *** *p* < 0.001, ** *p* < 0.01, and * *p* < 0.05.

## 3. Results

### 3.1. Participant Characteristics

The clinical characteristics are summarized in Table 1. A total of 74 women were selected, including 40 women experiencing normal pregnancies and 34 women experiencing GDM pregnancies. Moreover, 37 normal and 18 GDM participants had available data from two trimesters. The pregnant women who were complicated by GDM were significantly older (31.08 ± 3.024 vs. 33.30 ± 3.643 y, *p* = 0.015; 31.08 ± 2.944 vs. 33.62 ± 3.455 y, *p* = 0.001) and had a higher *p*-BMI (21.03 ± 2.287 vs. 22.70 ± 3.503 kg/m^2^, *p* = 0.03; 20.88 ± 2.281 vs. 22.93 ± 3.249 kg/m^2^, *p* = 0.003), as well as higher second-trimester TCHO (5.40 ± 0.745 vs. 6.25 ± 1.140 mmol/L, *p* = 0.006) and LDL levels (2.57 ± 0.505 vs. 3.15 ± 0.913 mmol/L, *p* = 0.015), than the women in the control group. There were also significant differences in the OGTT results. Other clinical factors were similar between groups and showed no significant differences.

### 3.2. Comparison of Maternal SCFA Levels

**Figure 1:**
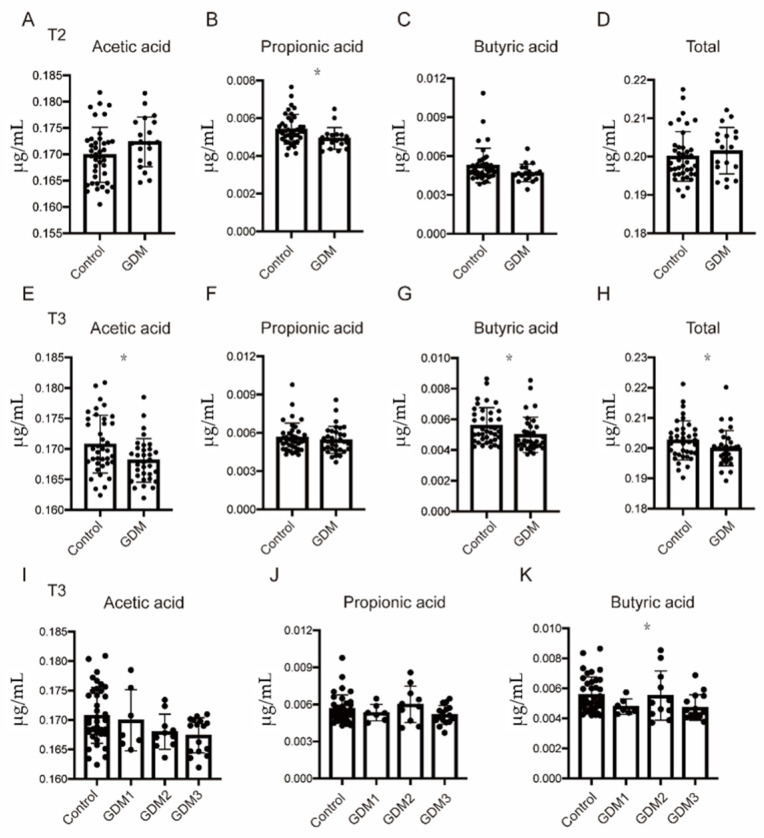
The levels of three dominant SCFAs and total SCFA were decreased among GDM pregnancies. (**A**–**D**) Circulating levels of acetic, propionic, butyric, and total acids in T2. (**E**–**H**) Circulating levels of acetic, propionic, butyric, and total acids in T3. (**I**–**K**) Circulating levels of acetic, propionic, and butyric acid among the control and GDM subtypes in T3. Data are presented as the mean ± SD. * *p* < 0.05, significant difference vs. control group.

The three main SCFA profiles (acetic acid, propionic acid, and butyric acid) in the GDM group differed from those in the normal group. As shown in Figure 1B, propionate levels were significantly decreased in women with GDM in T2. Similar results were obtained in T3, as manifested by significantly lower butyric acid, acetic acid, and total SCFA values in the GDM group (Figure 1E,G,H). Propionic acid exhibited the same trend, but no significant differences were observed. Furthermore, no differences found with respect to the other SCFAs (isobutyric acid, valeric acid, isovaleric acid, and hexanoic acid) in either trimester (Supplementary Figure S1A–H), and we excluded these SCFAs from subsequent analyses.

Given that abnormal glucose levels at different OGTT time points have disparate effects on pregnancy outcomes [25,26], we categorized women with GDM into three subgroups. The third trimester acetate and butyrate contents were obviously lower in the GDM subtype 3 group than in the normal group (Figure 1I,K). No such differences were found in T2 (Supplementary Figure S1I–K). Given that overweight/obesity was the cofounding factor affecting SCFA levels, participants were further stratified based on the BMI recommendations of the Group of China Obesity Task Force of the Chinese Ministry of Health. Only acetic acid was significantly decreased in the overweight/obese GDM group compared to the normal weight control group in T3 (Supplementary Figure S2).

### 3.3. Correlation Analysis between Circulating SCFAs and Clinical Indicators

**Figure 2:**
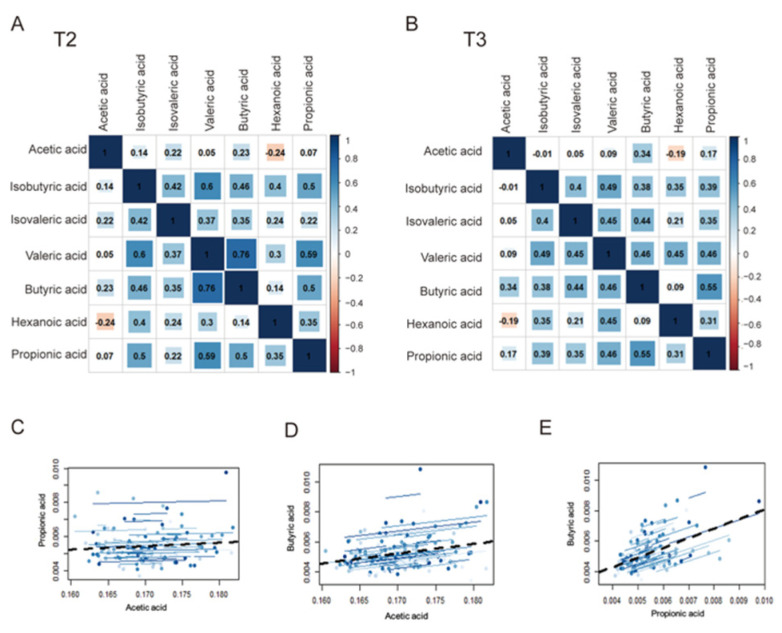
Relationships between SCFAs. (**A**,**B**) Correlation analysis between SCFAs in all pregnancies in T2 and T3. (**C**–**E**) Dynamic trajectory analysis between butyric acid and acetic or propionic acid in all pregnancies.

We performed correlation analysis among acetic, propionic, and butyric acids during the two studied trimesters. Butyric acid was positively correlated with propionic acid (r = 0.496, *p* < 0.001; r = 0.550, *p* = 0.001) and acetic acid (r = 0.230, *p* = 0.082; r = 0.338, *p* = 0.004) during both T2 and T3 (Figure 2A,B). We also observed positive correlations between propionic and acetic acids, although no significant differences were observed (r = 0.067, *p* = 0.616; r = 0.168, *p* = 0.163). Therefore, we performed dynamic trajectory analysis to comprehensively analyze the T2 and T3 datasets. Butyric acid was still significantly positively correlated with acetic acid (r = 0.275, *p* = 0.044) and propionic acid (r = 0.522, *p* < 0.001), indicating that the tendency observed in T2 could be sustained until T3 (Figure 2D,E).

**Figure 3:**
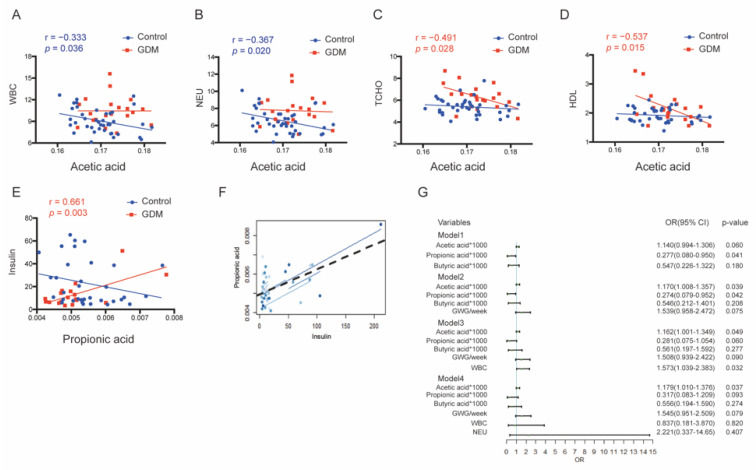
Correlation analysis between acetic, propionic, and butyric acid contents and clinical indicators in T2. (**A**–**D**) Associations between acetic acid and WBC, NEU counts, TCHO, or HDL levels in either group. (**E**,**F**) Associations between propionic acid and insulin levels. (**G**) Comparisons of GDM diagnostic models including acetic, propionic, butyric acid, and other parameters. WBC, white blood cell; NEU, neutrophil; TCHO, total cholesterol; HDL, high-density lipoprotein.

We then conducted correlation analysis between the dominant SCFAs (acetic, propionic, and butyric acids) and maternal inflammatory factors, glucolipid metabolism indicators, and maternal–neonatal demographic factors. The three SCFAs were negatively correlated with WBC and NEU counts in normal pregnancies, with more obvious correlations between acetic acid and the WBC (r = −0.333, *p* = 0.036) or NEU counts (r = −0.367, *p* = 0.020) (Figure 3A,B). Negative tendencies were also observed between butyric acid and WBC counts (r = −0.245, *p* = 0.065) in T2 and between propionic acid and the WBC (r = −0.286, *p* = 0.081) in T3, although no significant differences were observed (Supplementary Figure S3A–D).

**Figure 4:**
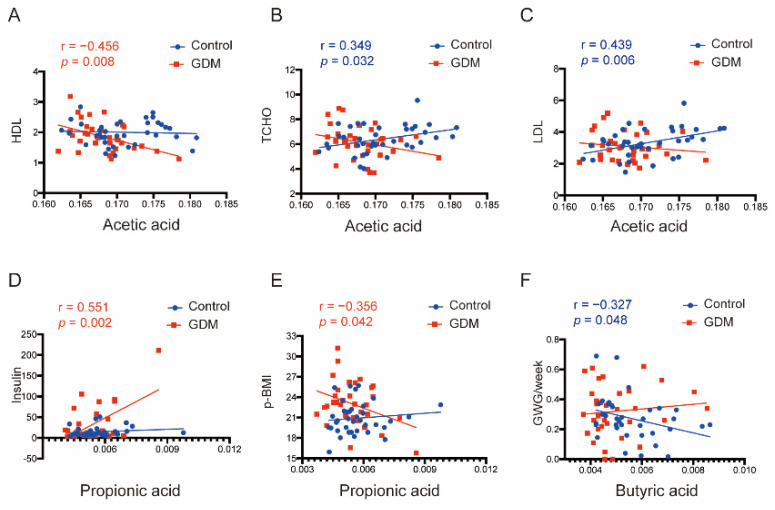
Correlation analysis between acetic, propionic, and butyric acid contents and clinical indicators in T3. (**A**–**C**) Associations between acetic acid and WBC, NEU counts or LDL level in either group. (**D**,**E**) Associations between propionic acid and insulin level or *p*-BMI in either group. (**F**) Associations between butyric acid and GWG ratio in either group.

In glucolipid-related analysis, acetic acid in GDM pregnancies was significantly inversely correlated with TCHO (r = −0.491, *p* = 0.028) and HDL levels (r = −0.537, *p* = 0.015) in T2 (Figure 3C,D), and the latter association was maintained until T3 (HDL: r = −0.456, *p* = 0.008) (Figure 4A). Conversely, in normal pregnancies, acetic acid was strongly positively correlated with the TCHO (r = 0.349, *p* = 0.032) and LDL levels in T3 (r = 0.439, *p* = 0.006) (Figure 4B,C). There were also significant positive correlations between propionic acid and insulin levels in GDM during both trimesters (r = 0.661, *p* = 0.003 in T2 and r = 0.551, *p* = 0.002 in T3, Figure 3E and Figure 4D). Notably, the relationship in the mid-trimester acted as a predictor for the relationship in the third trimester according to dynamic trajectory analysis (r = 0.671, *p* = 0.006, Figure 3F).

Finally, propionic acid was negatively related to *p*-BMI in GDM (r = −0.356, *p* = 0.042). Butyric acid exhibited a negative correlation with *p*-BMI but without significant differences (r = −0.301, *p* = 0.084). Notably, butyric acid was significantly inversely related to GWG per week before GDM diagnosis (r = −0.327, *p* = 0.048) in normal pregnancies (Figure 4E,F and Figure S3E).

**Figure 5:**
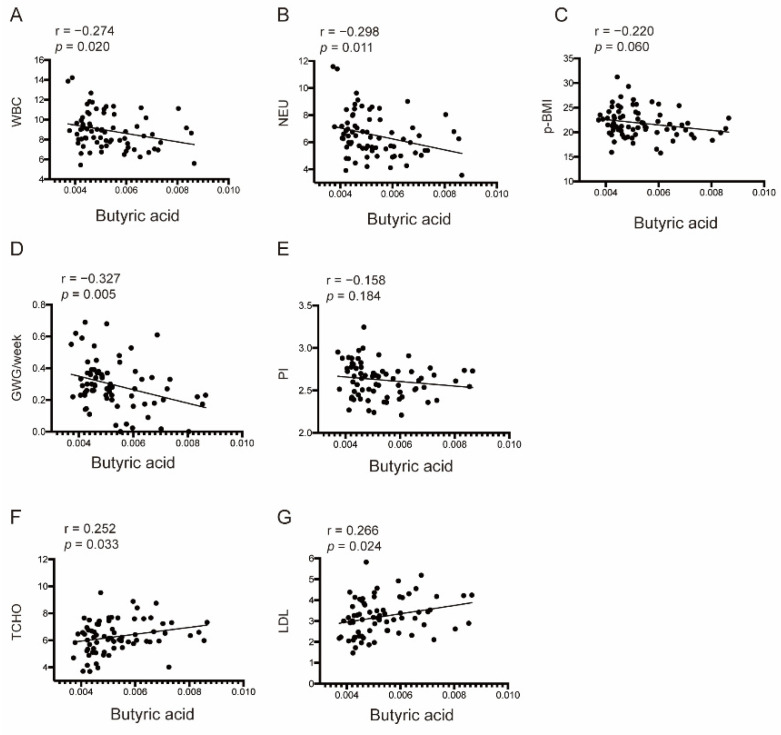
Butyric acid was a closely related clinical indicator in T3 among all pregnancies. (**A**–**G**) Associations between butyric acid and WBC counts, NEU counts, *p*-BMI, GWG ratio, PI, TCHO, and LDL levels. WBC, white blood cell; NEU, neutrophil; p-BMI, pre-pregnancy BMI; GWG, gestational weight gain; PI, Ponderal Index; TCHO, total cholesterol; LDL, low-density lipoprotein.

In all pregnancies, butyric acid was closely associated with most parameters, especially in T3. As shown in Figure 5, butyric acid was significantly negatively correlated with WBC counts, NEU counts, and GWG per week before GDM diagnosis but positively correlated with TCHO and LDL levels. A negative tendency was also observed between butyric acid and *p*-BMI and PI. Other correlation findings, such as the negative correlations between acetic, propionic, or butyric acid and either fasting or OGTT glucose levels, are summarized in Supplementary Table S2, although no obvious differences were found.

According to multivariable linear regression analysis adjusted for *p*-BMI, acetic acid was still significantly inversely related to TCHO in women with GDM (β = −0.0198, *p* = 0.007), and butyric acid was inversely related to WBC counts in normal pregnancies in T2 (β = −0.0014, *p* = 0.007). According to simple correlation analysis, the variables with weak associations, such as propionic and insulin levels (β < 0.0001, *p* = 0.003) and propionic and butyric levels with GWG per week before OGTT diagnosis (β = −0.0025, *p* = 0.022; β = −0.0038, *p* = 0.057), became pronounced in multiple regression analysis (Supplementary Table S3). Until the third trimester, butyric acid was also statistically negatively correlated with WBC counts in normal pregnancies, similar to previous results (β = −0.0016, *p* = 0.047), and accompanied by reduced TG (β = −0.0011, *p* = 0.016) and HDL levels (β = −0.0029, *p* = 0.037) (Supplementary Table S3).

### 3.4. Determination of Parameters Influencing GDM Occurrence

We next analyzed whether acetic, propionic, and butyric acids could be used alone or in combination with other parameters in T2 to distinguish between GDM and normal pregnancies. Propionic acid contributed to GDM incidence the most (Figure 3G); therefore, models including propionic acid were superior to other models (Supplementary Table S4). WBC counts were also identified as an important indicator. We found that the diagnostic model including acetic, propionic, and butyric acids and WBC counts was optimal, and a predictive value of 0.368 was identified as the best cutoff ratio (AUC = 0.823, *p* = 0.031, sensitivity = 83.3%, specificity = 79.5%) (Supplementary Table S4).

### 3.5. GPR41/43 Expression Was Decreased, and HDAC Expression Was Increased, Accompanied by Disrupted Glucose Metabolism in GDM Placentae

**Figure 6:**
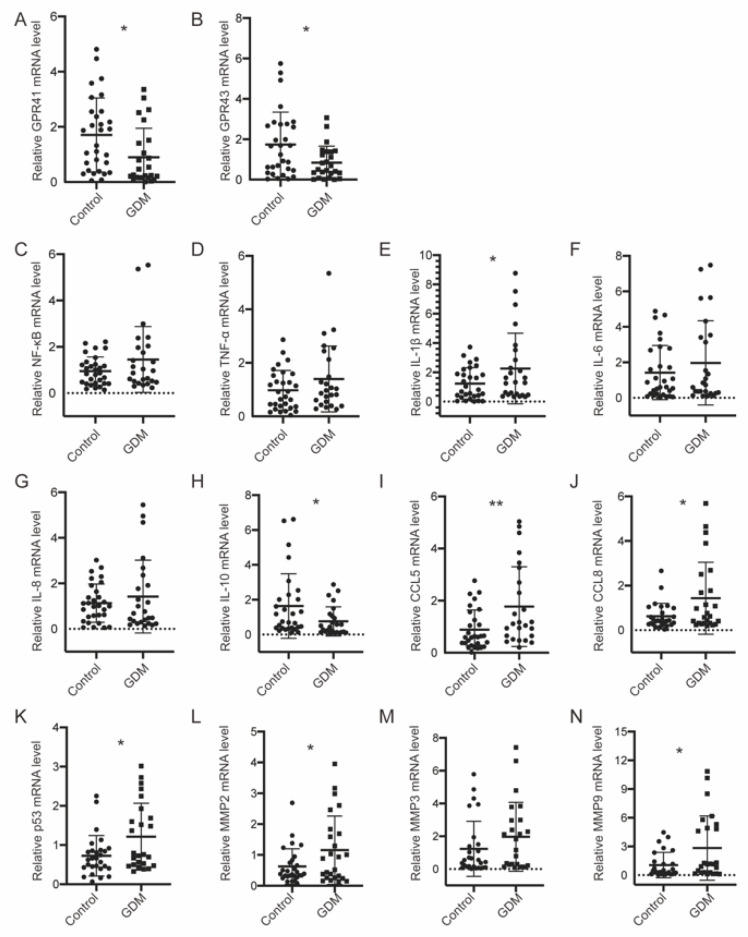
GPR41 and GPR43 were decreased, accompanied by enhanced proinflammatory responses in GDM pregnancies. (**A**,**B**) mRNA levels of GPR41/43 measured by real-time PCR. (**C**–**N**) mRNA levels of cytokines measured by real-time PCR. Data are presented as the mean ± SD. * *p* < 0.05, ** *p* < 0.01, significant difference vs. control group. GPR41, G-protein-coupled receptors 41; GPR43, G-protein-coupled receptors 43.

**Figure 7:**
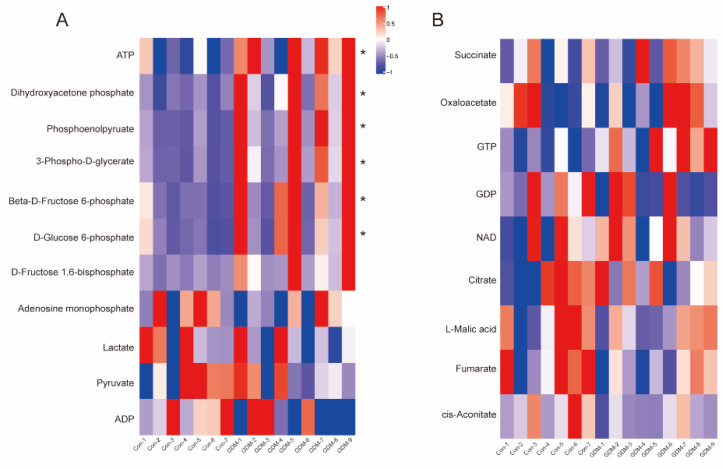
Placental glycolysis and the TCA cycle were compromised under hyperglycemic conditions. (**A**,**B**): Heatmaps of the detected intermediates in glycolysis and the TCA cycle, respectively. ATP, adenosine triphosphate; ADP, adenosine diphosphate; GTP, guanosine triphosphate; GDP, guanosine diphosphate; NAD, nicotinamide adenine dinucleotide. * *p* < 0.05.

Acting as signaling molecules, SCFAs function by coupling with GPR41/43 or HDACs to modulate physical activities. We detected the mRNA expression level in placentae and observed that both receptors were significantly upregulated in the normal group (Figure 6A,B), whereas HDAC4, HDAC8, and HDAC9 were significantly increased in the GDM group (Supplementary Figure S4). Concomitant with this, anti-inflammatory IL−10 was significantly decreased in GDM, and other proinflammatory molecules were increased (Figure 6C–N). The overall metabolic status was evaluated by examining the key intermediates of glycolysis and the TCA cycle through targeted metabolomics. Upstream mediators, such as D-glucose 6-phosphate, 3-phospho-D-glycerate, and beta-D-fructose 6-phosphate, were significantly increased in the GDM group (Figure 7A). The same trends were identified in downstream substances, including dihydroxyacetone phosphate and phosphoenolpyruvate. In addition, the GDM pregnancies were unique in their ability to generate ATP through high rates of glycolysis; more ATP production was observed in the GDM group. Concordantly, the metabolites participating in the TCA cycle were lower in the GDM group, although no significant differences were observed (Figure 7B).

## 4. Discussion

In this study, we compared the maternal circulating SCFA contents between GDM and normal pregnancies and observed that the three most abundant SCFAs (acetic, propionic, and butyric acids) were obviously decreased in GDM in both trimesters, in accordance with the levels observed in diabetic and obese pregnant women [10,14]. Compared to a single hyperglycemia value, GDM pregnancies combined with fasting and post-load abnormal glucose levels (subtype 3) were associated with exaggerated disruptions in the glucose metabolic balance and insulin sensitivity [27]. We also found the lowest butyric and acetic levels in the subtype 3 group in the third trimester. The inadequate production capacity did not compensate for the increasing insulin resistance and low-grade inflammation in susceptible populations [28], which may contribute to the initiation and development of GDM. Given that *p*-BMI was a confounding factor affecting SCFA levels, the participants were stratified based on *p*-BMI. No significant differences were found between normal and overweight/obese pregnancies in either the control or GDM group, indicating that GDM alone or in combination with overweight/obesity contributes to SCFA variations. Due to the smaller proportion and negligible differences in other SCFAs, we excluded these SCFAs from the subsequent analyses.

Close relationships between various circulating SCFAs are common and can also be found in human milk [29,30]. Considering that individual dependencies may mask one another, we determined the association between the SCFAs and clinical parameters in each group and among all of participants. In normal pregnancies, the fat deposition process is associated with an increase in the concentrations of TGs and other fatty acids, and we found positive correlations between acetate and TCHO/LDL levels in T3 [31]. Acetic acid serves as a primary substrate in cholesterol synthesis [32], exerting an antilipolytic effect by decreasing lipolysis and increasing fatty acid oxidation. The inhibitory effects also attenuated ectopic lipid accumulation by reducing lipid extravasation and benefitting maternal health [33]. However, we found that acetic acid was inversely related to TCHO and HDL levels in GDM, in contrast to findings in obese pregnancies that propionic acid is positively correlated with HDL levels. This finding may support the hypothesis that feedback regulation occurs under the influence of disturbed metabolic balance. Moreover, acetic and propionic acids play opposite roles in lipogenesis and hepatic cholesterogenesis [34]. A positive correlation between propionic acid and insulin levels was found in the two trimesters, both separately and combined. It has been further suggested that propionate can stimulate insulin secretion and protect β-cells from apoptosis induced by proinflammatory cytokines, which are transmitted by GPRs [18,35]. In addition, circulating propionic acid is positively related to insulin sensitivity as a result of the increased stimulation of glucose uptake accompanied by the suppression of lipogenesis [36,37].

In GDM pregnancies, the propionic and butyric acid contents in T3 were significantly negatively correlated with *p*-BMI, whereas in T2, acetic acid showed a positive correlation with *p*-BMI, although no significant differences were found. The same findings have been observed in obese pregnant women, who are more capable of oxidizing sugars than lipids and constitute a portion of the GDM population [38]. Potential mechanisms may include the antiobesity effects of propionic and butyric acids via the inhibition of energy intake mediated via GLP−1 and PYY [9,39], whereas acetic acid formulation has been associated with body weight gain. In addition, all of the women diagnosed with GDM in our hospital are directed to lifestyle treatments, such as diet and/or exercise guidance, which have the potential to improve their metabolic status in the last trimester. We did not find any associations between SCFAs and GWG, with the exception of an inverse correlation between butyric acid and GWG per week before GDM diagnosis [10]. Combined with the beneficial function of butyric acid, this finding suggests that the predisposing weight gain before GDM diagnosis is associated with GDM onset and that sustained weight gain is related to metabolic complications for mothers later in life.

The anti-inflammatory function of SCFAs was obvious in our study and was manifested by their negative correlations with the WBC and NEU count. Consistent with this, GPR41/43 expression was reduced, and HDAC expression was upregulated, weakening the suppression of proinflammatory cytokines in GDM placentae. Previous studies demonstrated that SCFA supplementation could inhibit LPS- and/or TNF-α induced inflammation through GPR41, GPR43, or HDACs in human umbilical vein endothelial cells (HUVECs) and in human renal cortical epithelial cells [40,41]. Notably, the microbiota and its metabolites play pivotal roles in immune–metabolic activities, and we observed activated glycolysis in the placentae of GDM pregnancies [42]. Glycolytic-related genes, such as PGK2 and GCK, were also found to be upregulated in adipose tissue samples from women with GDM [21]. Concomitantly, metabolites tended to be decreased in the TCA cycle, which can be explained in terms of the biological similarities between placentae and malignant tumors, such as aerobic glycolysis [43]. The placenta plays an important role in the maternal–fetal interface, and alterations in its immune–metabolic activities may influence fetal growth and development. However, we only found a negative tendency of associations between the SCFAs and neonatal demographic factors at birth, similar to observations in human milk [30]. A consistent negative association was found from 3 months of age; therefore, follow-up studies are necessary. Butyrate constitutes a major energy source for intestinal epithelial cells and metabolic responses [44]. It promotes intestinal epithelial cell growth and enhances epithelial barrier function, avoiding “leaky gut” and GDM onset [45]. We found that butyric acid is closely associated with most clinical information in all pregnancies and that it exerted antidiabetic effects, which is consistent with previously reported results.

There were no significant differences between the normal and overweight/obese pregnancies in terms of acetic, propionic, or butyric acids in either group. We obtained similar results in the multivariable linear regression analysis adjusted for *p*-BMI. In logistic analysis after correcting for *p*-BMI, propionate was found to contribute to the incidence of GDM the most. Considering their close relationships with the WBC counts in T2, we incorporated the WBCs into the diagnostic model and obtained optimal efficiency.

Few studies have assessed the correlations between maternal SCFAs and clinical indicators complicated by GDM or their roles as signaling molecules affecting the functions of distal organs, such as the placenta, through GPRs or HDACs. We stratified pregnancies based on OGTT results and *p*-BMI to identify the primary effects of glucose intolerance on SCFA contents. Combined correlation analysis (single and dynamic trajectory analysis), multivariable regression analysis, and logistic analysis enabled us to confirm the influential factors associated with GDM development. Notably, we detected SCFA contents in maternal circulation rather than in feces. This is more representative, as approximately 95% of colonic SCFAs are absorbed into the blood and are connected to metabolic health [36]. The description of placental immune–metabolic responses establishes their connections with distal placenta through GPR41, GPR43, and HDACs. Diet seems to play a key role in regulating SCFA levels during pregnancy. Studies have demonstrated that a high-fat, low-fiber diet in GDM pregnancies could alter the composition of SCFA-producing microbiota and could lead to excessive SCFA generation [45]. However, this would lead to the overflow of free fatty acids, causing adverse health effects if SCFA elevation exceeds the normal lipid storage capacity, resulting in a positive energy balance [46]. Other pathways related to SCFA production, such as fatty acid oxidation, also may have influenced the current results. Therefore, further studies with larger sample sizes and diet questionnaires are needed. SCFA contents in the umbilical cord are dominant in evaluations of newborn development and positively correlated with maternal circulation levels [11]. Maternal SCFA levels and their associations with neonatal anthropometrics may reflect fetal growth conditions, aiding in the interpretation of the influences of maternal metabolic status on the fetus.

Briefly, maternal circulating SCFAs (especially acetic, propionic, and butyric acids) were found to be closely related to clinical indicators in GDM pregnancies and may exert favorable effects on physiological activities directly or through GPRs and HDACs, providing more evidence with respect to their potential therapeutic targets for GDM pregnancies and basic research.

## 5. Conclusions

Maternal circulating acetic, propionic, and butyric acids showed potential antidiabetic and anti-inflammatory effects in GDM women. They influence placental immunometabolism and fetal development at birth through GPRs or HDACs and may be therapeutic targets for GDM pregnancies.

## Figures and Tables

**Table 1:** Characteristics of the study population in the second and third trimesters.

	Control (*n* = 40,T2)	GDM (*n* = 20,T2)	*p*-Value
Age (years)	31.08 ± 3.024	33.30 ± 3.643	0.015 *
Gestational week at sampling (Weeks)	25.18 ± 0.984	25.50 ± 0.889	0.218
*p*-BMI (kg/m^2^)	21.03 ± 2.287	22.70 ± 3.503	0.03 *
GWG (kg)	13.29 ± 3.170	12.62 ± 4.821	0.58
GLU 0 h (mmol/L) ^a^	4.62 ± 0.274	5.17 ± 0.578	<0.0001 ****
GLU 1 h (mmol/L) ^a^	7.61 ± 1.245	10.26 ± 1.389	<0.0001 ****
GLU 2 h (mmol/L) ^a^	6.07 ± 1.048	8.86 ± 1.327	<0.0001 ****
AUC	12.96 ± 1.582	17.28 ± 2.015	<0.0001 ****
Glucose-T2 (mmol/L)	4.21 ± 0.649	4.87 ± 0.478	<0.0001 ****
TG-T2 (mmol/L)	2.12 ± 0.655	2.24 ± 1.005	0.607
TCHO-T2 (mmol/L)	5.40 ± 0.745	6.25 ± 1.140	0.006 **
HDL-T2 (mmol/L)	1.92 ± 0.241	2.15 ± 0.516	0.077
LDL-T2 (mmol/L)	2.57 ± 0.505	3.15 ± 0.913	0.015 *
	Control (*n* = 38, T3)	GDM (*n* = 34, T3)	*p*-value
Age (years)	31.08 ± 2.944	33.62 ± 3.455	0.001 **
Gestational week at sampling (Weeks)	35.24 ± 1.051	35.26 ± 1.16	0.915
*p*-BMI (kg/m^2^)	20.88 ± 2.281	22.93 ± 3.249	0.003 **
GWG (kg)	13.17 ± 3.209	11.58 ± 4.349	0.08
GLU 0 h (mmol/L) ^a^	4.59 ± 0.281	5.22 ± 0.571	<0.0001 ****
GLU 1 h (mmol/L) ^a^	7.63 ± 1.235	10.16 ± 1.266	<0.0001 ****
GLU 2 h (mmol/L) ^a^	6.13 ± 1.037	8.66 ± 1.334	<0.0001 ****
AUC	12.99 ± 1.592	17.10 ± 1.805	<0.0001 ****
Glucose-T3 (mmol/L)	4.57 ± 0.491	4.83 ± 0.679	0.068
TG-T3 (mmol/L)	2.72 ± 0.790	2.83 ± 1.050	0.596
TCHO-T3 (mmol/L)	6.40 ± 1.105	6.10 ± 1.350	0.293
HDL-T3 (mmol/L)	2.00 ± 0.396	1.83 ± 0.495	0.108
LDL-T3 (mmol/L)	3.32 ± 0.880	3.08 ± 0.906	0.251
FBW (g)	3297.76 ± 305.978	3266.76 ± 379.046	0.702
Height (cm)	50.08 ± 0.997	49.88 ± 1.174	0.445
PI (kg/m^3^)	2.62 ± 0.207	0.178 ± 0.201	0.999
Head circumference (cm)	34.35 ± 0.645	34.02 ± 0.856	0.059
Placenta weight (g)	548.57 ± 69.25	583.38 ± 102.97	0.126
Placenta volume (cm^3^)	742.42 ± 130.33	784.97 ± 213.29	0.307
FPR	0.168 ± 0.022	0.178 ± 0.046	0.259

^a^: Results of the 75 g oral glucose tolerance test are expressed as mean ± SD. GWG, gestational weight gain; GLU, glucose; AUC, area under curve; TG, total triglycerides; TCHO, total cholesterol; HDL, high-density lipoprotein; LDL, low-density lipoprotein; FBW, fetal birth weight; PI, ponderal index; FPR, fetal–placenta ratio, **** *p* < 0.0001, ** *p* < 0.01 and * *p* < 0.05.

## Data Availability

Not applicable.

## Supplementary Materials

The following supporting information can be downloaded at: https://www.mdpi.com/article/10.3390/nu14183727/s1, Figure S1: The levels of other SCFAs were of no significant differences between control and GDM pregnancies; Figure S2: Comparisons among the three dominant SCFAs stratified by *p*-BMI; Figure S3: Correlation analysis between acetic, propionic, and butyric acid contents and clinical indicators in T2 and T3; Figure S4: HDACs were increased in GDM pregnancies; Table S1: Primer sequences of RT-PCR used in this study; Table S2: Correlation analysis between SCFA (acetic, propionic, and butyric acid) levels and clinical parameters in T2 and T3 after adjusting for *p*-BMI; Table S3: Multivariable linear regression analysis between SCFAs (acetic, propionic, and butyric acid) and clinical parameters in T2 and T3 after adjusting for *p*-BMI; Table S4: Capacity for GDM determination using acetic, propionic, and butyric acids alone and in combination.
