# Supplementary material for: Composition of Maternal Circulating Short-Chain Fatty Acids in Gestational Diabetes Mellitus and Their Associations with Placental Metabolism
Source: Nutrients. 2022 Sep 9;14(18):3727. doi: 10.3390/nu14183727 (PMC9505713; doi:10.3390/nu14183727)
Supplement: Supplementary file 1 [file nutrients-14-03727-s001.zip › nutrients-1886243-supplementary.pdf]

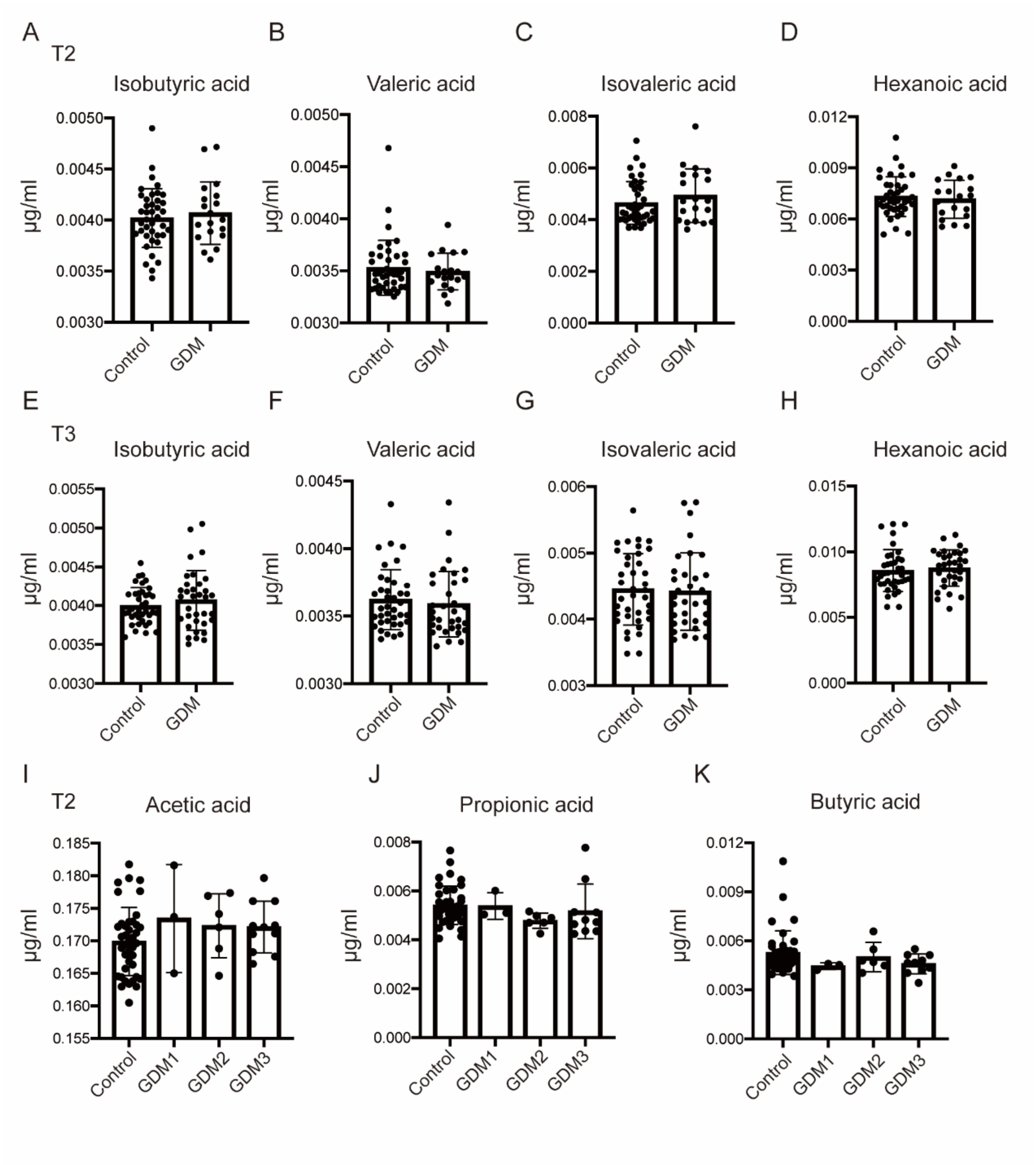

Figure S1. The levels of other SCFAs were of no significant differences between control and GDM pregnancies. (A-H) The circulating levels of isobutyric, valeric, isovaleric and hexanoic acid in T2 (A-D) and T3 (E-H). (I-K) The circulating levels of acetic, propionic, and butyric acid among the control and GDM subtypes in T2. The data are presented as the mean ± SD.

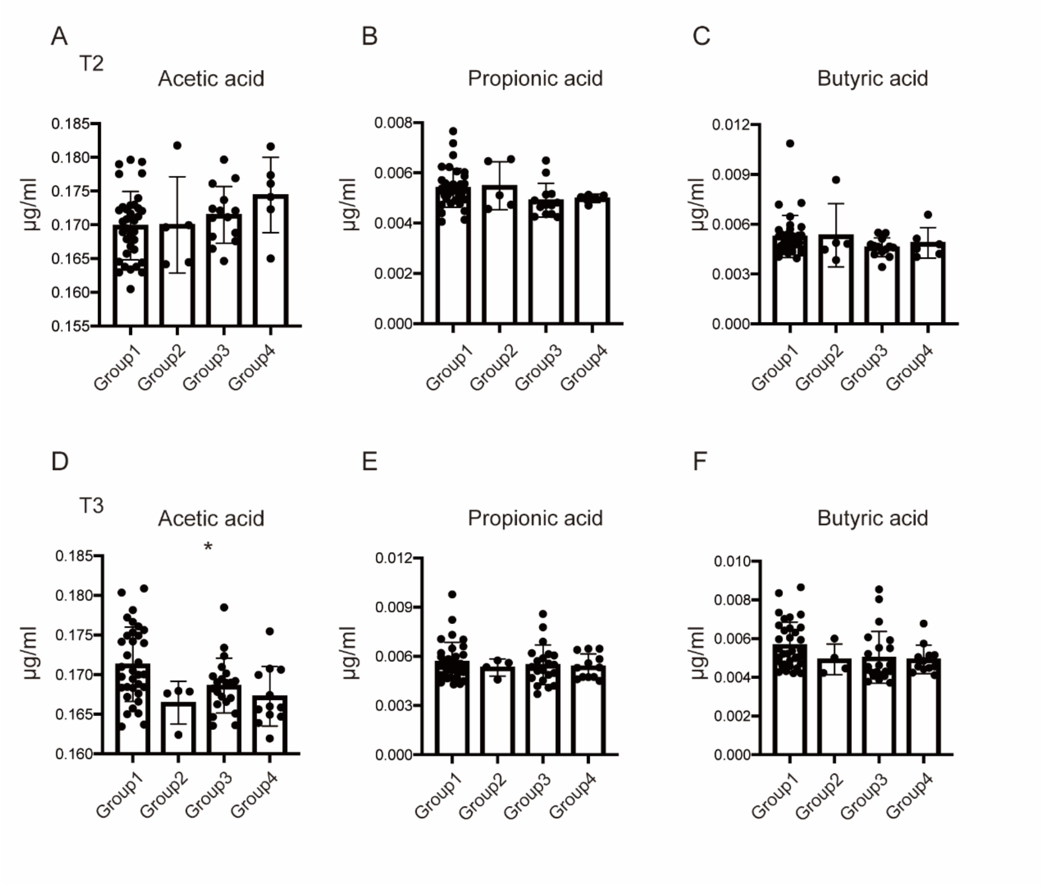

Figure S2. Comparisons among the three dominant SCFAs stratified by p-BMI. (A-F) Maternal circulating levels of acetic, propionic and butyric acid in T2 (A-C) and T3 (D-F). The data are presented as the mean ± SD. **p*< 0.05 indicates a significant difference vs Group1. Group1: Control group of normal weight; Group2: Control group of overweight/obesity; Group3: GDM of normal weight; Group4: GDM of overweight/obesity.

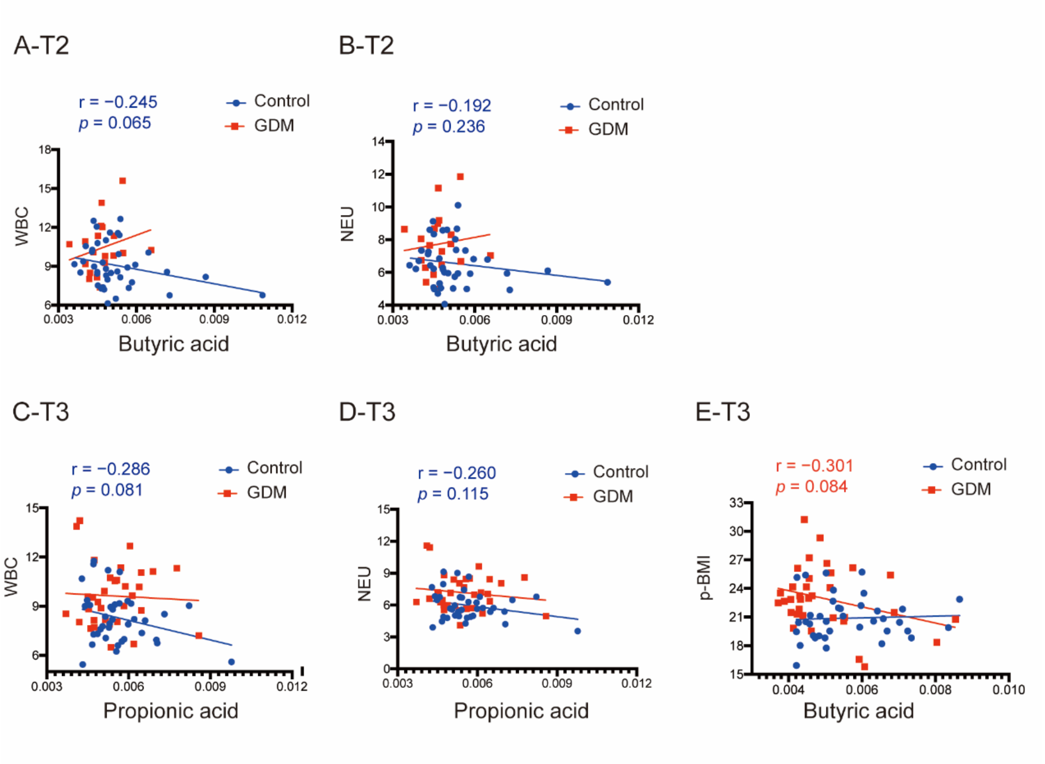

Figure S3. Correlation analysis between acetic, propionic and butyric acid contents and clinical indicators in T2 and T3. (A-B) The associations between acetic acid and WBC or NEU counts in either group. (C-D) The associations between propionic acid and WBC or NEU counts in either group. (E) The associations between butyric acid and p-BMI in either group.

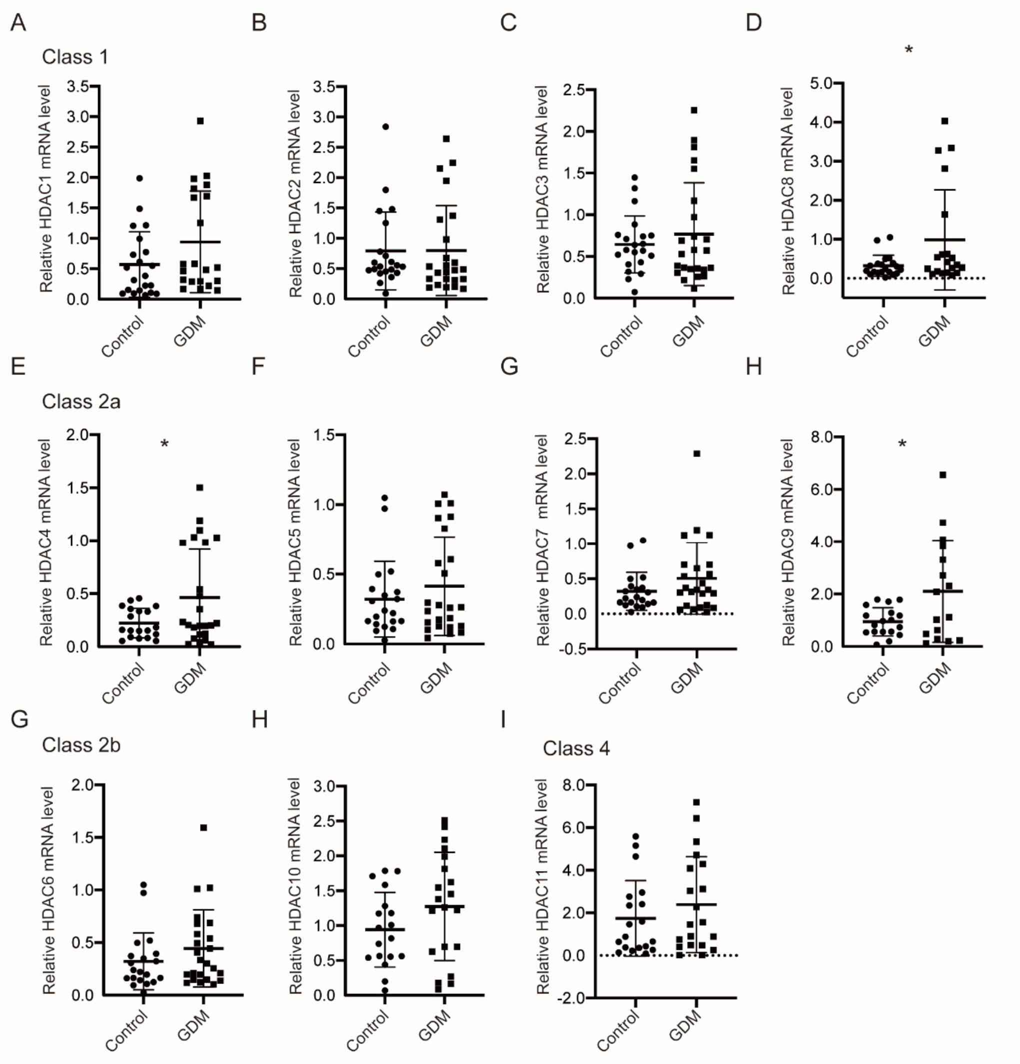

Figure S4. HDACs were increased in GDM pregnancies. (A-K) The mRNA levels of HDACs were measured by real-time PCR. The data are presented as the mean ± SD. *$p < 0.05$ as significant difference vs. control group.

Table S1. Primer Sequences of RT-PCR used in this study.

Gene	Species	Forward	Reverse
GPR41	Human	TGGTTCGTCTTCTCGGTGT	TCAGGTTGAGCAGGAGCA
GPR43	Human	GAGCCTGGAGATGAGCAG	GGGGAGACAAGACAACACA
IL-1β	Human	TTGAGTCTGCCCAGTTCC	TTTCTGCTTGAGAGGTGCT
IL-6	Human	CAATAACCACCCCTGACC	GCGCAGAATGAGATGAGTT
IL-8	Human	GTGCTGTGTTGAATTACGGA	TTGACTGTGGAGTTTTGGC
IL-10	Human	ACCAAGACCCAGACATCAA	CATTCTTCACCTGCTCCAC
CCL5	Human	GTGTGCCAACCCAGAGA	GGATAGTGAGGGGAAGCC
CCL8	Human	GATGGAGAGCACCAGCA	TGGAAGAGAGCCAAGGG
NF-κB	Human	CCCATCTTTGACAATCGTGC	CTGGTCCCGTGAAATACACC
TNFα	Human	CACCACTTCGAAACCTGGGA	AGGAAGGCCTAAGGTCCACT
P53	Human	CCAGATGAAGCTCCCAGA	GGGAAGGGACAGAAGATGA
MMP2	Human	CGCCTTTAACTGGAGCAAA	AGGTTATCGGGGATGGC
MMP3	Human	TTTTCTCCTGCCTGTGCT	TTCACGCTCAAGTTCCCT
MMP9	Human	ACGCAGACATCGTCATCC	CCAGGGACCACAACTCG
HDAC1	Human	ATATCGTCTTGGCCATCCTG	GGAGCGGGTAGTTAACAGGCA
HDAC2	Human	GGTGCTGGAAAAGGCAAATA	ACGGATTGTGTAGCCACCTC
HDAC3	Human	TGGCTTCTGCTATGTCAACG	TCTCTGCCCCGACTTCATAC
HDAC4	Human	CCCTGTTACGCAGGAAAGAC	AGGATGGCGATGTGTAGAGG
HDAC5	Human	CGCAAGGATGGGACTGTTAT	ATGTTGGGCAGAGAAGGAGA
HDAC6	Human	GGGCCACAGAGAAAATGAAA	CCACAGCCACAGAGAGTTGAAA
HDAC7	Human	CAGCTTTTTGCCTCCTGTTC	GGCGTGCTGCTACTACTTGG
HDAC8	Human	CGGAATGTGCAAAGTAGCAA	CCAACATCAGACACGTCACC
HDAC9	Human	CAGCAACAAATCCAGAAGCA	AGGAAGCTGCTGTTCTCTGC
HDAC10	Human	CTCACTGGAGCTGTGCAAAA	CGGTGCCAGGAGAAGTAAAG
HDAC11	Human	CTCCAGGGCTACCATCATTG	CCTGGAGGGATTTCTTGATG
18S	Human	AGAAACGGCTACCACATCCA	TACAGGGCCTCGAAAGAGTC

Table S2. Correlation analysis between SCFAs (acetic, propionic and butyric acid) levels and clinical parameters in T2 and T3 after adjusting for p-BMI.

Variables	Acetic acid				Propionic acid				Butyric acid			
	T2		T3		T2		T3		T2		T3	
	r	*P*-value	r	*P*-value	r	*P*-value	r	*P*-value	r	*P*-value	r	*P*-value
**GDM**												
GWG/week	-0.191	0.419	-0.047	0.796	0.250	0.301	-0.067	0.712	0.361	0.129	0.013	0.944
Glucose	0.223	0.344	0.010	0.956	0.373	0.116	0.100	0.585	0.004	0.988	-0.083	0.645
p-BMI	0.419	0.067	-		0.186	0.446	-		0.167	0.493	-	
GLU0	-0.008	0.974	-		0.380	0.109	-		-0.075	0.761	-	
GLU1	-0.219	0.355	-		-0.382	0.107	-		-0.274	0.257	-	
GLU2	-0.251	0.287	-		-0.111	0.652	-		-0.301	0.211	-	
AUC	-0.234	0.320	-		-0.243	0.316	-		-0.300	0.213	-	
GWG	-		-0.083	0.648	-		0.164	0.363	-		0.157	0.376
CRP	-		0.083	0.653	-		-0.144	0.432	-		-0.063	0.729
Height	-		0.264	0.137	-		-0.139	0.439	-		0.148	0.402
FBW	-		0.206	0.250	-		-0.248	0.165	-		-0.055	0.757
PI	-		0.080	0.660	-		-0.258	0.148	-		-0.226	0.200
Placental weight	-		0.133	0.534	-		-0.063	0.768	-		-0.289	0.171
Volume	-		0200	0.274	-		0.082	0.654	-		0.065	0.718
FPR	-		0.041	0.851	-		0.080	0.710	-		-0.048	0.823
**Control**												
GWG/week	-0.134	0.416	0.065	0.702	-0.107	0.518	0.122	0.473	-0.198	0.226	0.041	0.808

Glucose	-0.237	0.151	-0.097	0.562	0.096	0.565	-0.016	0.923	0.032	0.851	0.090	0.588
p-BMI	-0.186	0.250	-		-0.02	0.898	-		-0.045	0.781	-	
GLU0	-0.031	0.849	-		-0.126	0.439	-		-0.053	0.743	-	
GLU1	0.116	0.475	-		0.050	0.760	-		-0.113	0.489	-	
GLU2	-0.008	0.959	-		0.165	0.309	-		0.015	0.926	-	
AUC	0.086	0.597	-		0.083	0.610	-		-0.088	0.588	-	
GWG	-		-0.087	0.602	-		0.064	0.704	-		-0.244	0.140
CRP	-		0.150	0.421	-		-0.054	0.774	-		0.161	0.386
Height	-		0.083	0.621	-		0.267	0.105	-		-0.026	0.875
FBW	-		0.028	0.866	-		0.104	0.536	-		-0.102	0.543
PI	-		-0.032	0.847	-		-0.074	0.661	-		-0.110	0.510
Placental weight	-		0.061	0.728	-		0.171	0.326	-		0.319	0.062
Volume	-		0.158	0.345	-		0.090	0.589	-		0.212	0.201
FPR	-		0.003	0.986	-		0.110	0.536	-		0.364	0.034
**All**												
GWG/week	-0.083	0.532	0.104	0.391	-0.027	0.843	0.057	0.642	-0.129	0.335	0.097	0.423
Glucose	-0.0006	0.997	-0.121	0.317	0.059	0.661	0.008	0.948	-0.069	0.608	-0.063	0.603
p-BMI	0.105	0.424	-		0.017	0.897	-		-0.044	0.743	-	
GLU0	0.110	0.401	-		0.005	0.970	-		-0.153	0.246	-	
GLU1	0.164	0.212	-		-0.207	0.117	-		-0.244	0.062	-	
GLU2	0.113	0.390	-		-0.107	0.418	-		-0.193	0.143	-	
AUC	0.156	0.233	-		-0.174	0.189	-		-0.241	0.066	-	
GWG	-		-0.010	0.935	-		0.131	0.278	-		0.033	0.782
CRP	-		-0.067	0.602	-		-0.138	0.281	-		-0.149	0.239
Height	-		0.186	0.121	-		0.082	0.496	-		0.083	0.487

FBW	-	0.118	0.328	-	-0.066	0.582	-	-0.063	0.601
PI	-	0.009	0.940	-	-0.155	0.196	-	-0.158	0.184
Placental weight	-	0.021	0.875	-	0.038	0.774	-	-0.014	0.917
Volume	-	0.123	0.312	-	0.070	0.562	-	0.087	0.469
FPR	-	-0.028	0.835	-	0.069	0.605	-	0.065	0.629

p-BMI, pre-pregnancy BMI; GWG, gestational weight gain; AUC, area under curve; CRP, C-reactive protein; FBW, Fetal birth weight; PI, Ponderal Index; FRP, fetal-placental ratio.

Table S3. Multivariable linear regression analysis between SCFAs (acetic, propionic and butyric acid) and clinical parameters in T2 and T3 after adjusting for p-BMI.

Variables	Acetic acid				Propionic acid				Butyric acid			
	T2		T3		T2		T3		T2		T3	
	β	*P*-value	β	*P*-value	β	*P*-value	β	*P*-value	β	*P*-value	β	*P*-value
**GDM**												
p-BMI	0.0007	0.058	0.0016	0.038	0.0001	0.290	0.0001	0.555	<0.0001	0.777	<0.0001	0.879
Glucose	-0.0025	0.338	0.0148	0.037	0.0002	0.631	0.0016	0.374	-0.0002	0.848	<0.0001	0.956
HbA1c	0.0037	0.476	-0.0001	0.162	0.0006	0.547	<0.0001	0.946	-0.0007	0.681	<0.0001	0.251
Insulin	-0.0002	0.221	-0.0120	0.068	<0.0001	0.677	-0.0010	0.638	<0.0001	0.757	0.0010	0.393
TG	0.0002	0.847	-0.0091	0.164	-0.0003	0.170	-0.0023	0.270	0.0003	0.432	-0.0023	0.173
TCHO	-0.0198	0.007	0.0265	0.146	-0.0025	0.097	0.0064	0.280	0.0006	0.715	0.0060	0.189
HDL	0.0171	0.016	-0.0585	0.080	0.0023	0.091	-0.0100	0.330	-0.0011	0.497	-0.0090	0.197
LDL	0.0220	0.008	-0.0236	0.175	0.0032	0.082	-0.0066	0.269	-0.0008	0.689	-0.0054	0.217
WBC	0.0025	0.460	0.0037	0.695	0.0002	0.791	-0.0008	0.418	0.0007	0.530	0.0005	0.880
NEU	-0.0034	0.427	-0.0058	0.606	-0.0004	0.613	0.0008	0.511	-0.0007	0.608	-0.0005	0.916
GWG/week	0.0014	0.824	0.0304	0.346	0.0020	0.195	0.0031	0.477	0.0004	0.837	-0.0007	0.966
CRP	-		-0.0001	0.822	-		<0.0001	0.495	-		<0.0001	0.928
GWG	-		-0.0008	0.315	-		-0.0001	0.358	-		0.0001	0.843
Birth weight	-		<0.0001	0.880	-		<0.0001	0.341	-		<0.0001	0.914
Height	-		0.0129	0.690	-		-0.0002	0.737	-		0.0015	0.886
FPR	-		0.2955	0.280	-		0.0288	0.211	-		0.0186	0.812
PI	-		0.0097	0.967	-		0.0009	0.171	-		0.0075	0.924
**Control**												

p-BMI	-0.0002	0.615	-0.0005	0.200	<0.0001	0.559	<0.0001	0.978	0.0001	0.448	-0.0001	0.393
Glucose	0.0008	0.720	0.0038	0.095	0.0010	0.002	0.0003	0.391	0.0008	0.165	0.0006	0.196
Insulin	-0.0001	0.334	0.0001	0.292	<0.0001	0.003	<0.0001	0.853	<0.0001	0.388	0.0001	0.009
TG	0.0027	0.397	0.0014	0.475	0.0005	0.254	<0.0001	0.988	0.0001	0.898	-0.0011	0.016
TCHO	0.0052	0.658	-0.0002	0.975	-0.0032	0.064	-0.0005	0.622	-0.0017	0.556	0.0020	0.118
HDL	-0.0100	0.534	0.0014	0.823	0.0032	0.173	0.0001	0.900	-0.0008	0.852	-0.0029	0.037
LDL	-0.0093	0.476	-0.0009	0.887	0.0037	0.056	0.0003	0.760	0.0025	0.448	-0.0022	0.116
WBC	-0.0012	0.507	0.0007	0.760	-0.0002	0.450	<0.0001	0.961	-0.0014	0.007	-0.0016	0.047
NEU-T2	<0.0001	0.990	-0.0004	0.893	0.0002	0.614	-0.0001	0.827	0.0014	0.035	0.0019	0.064
GWG/week	-0.0007	0.923	0.0020	0.787	-0.0025	0.022	0.0007	0.635	-0.0038	0.057	-0.0013	0.573
CRP	-		0.0001	0.819	-		<0.0001	0.960	-		<0.0001	0.888
GWG	-		-0.0005	0.200	-		-0.0001	0.281	-		-0.0001	0.359
Birth weight	-		<0.0001	0.966	-		<0.0001	0.461	-		<0.0001	0.369
Height	-		-0.0010	0.940	-		0.0019	0.472	-		0.0047	0.348
FPR	-		-0.0291	0.599	-		-0.0121	0.272	-		0.0065	0.705
PI	-		-0.0053	0.948	-		0.0114	0.479	-		0.0291	0.356
**All**												
p-BMI	0.0002	0.466	0.0001	0.602	<0.0001	0.682	<0.0001	0.650	<0.0001	0.826	-0.0001	0.423
Glucose-T2	0.0017	0.174	0.0035	0.019	0.0002	0.436	0.0001	0.761	-0.0001	0.821	0.0005	0.140
Insulin-T2	-0.0001	0.005	<0.0001	0.469	<0.0001	0.711	<0.0001	0.797	<0.0001	0.609	<0.0001	0.534
TG-T2	0.0019	0.088	0.0019	0.255	<0.0001	0.979	0.0002	0.511	0.0002	0.405	-0.0003	0.457
TCHO-T2	-0.0061	0.159	-0.0049	0.259	-0.0007	0.359	-0.0008	0.246	-0.0006	0.558	-0.0002	0.815
HDL-T2	0.0037	0.458	0.0043	0.408	0.0002	0.798	0.0005	0.505	-0.0001	0.953	-0.0008	0.521
LDL-T2	0.0063	0.207	0.0044	0.359	0.0006	0.479	0.0006	0.395	0.0007	0.594	0.0003	0.788
WBC-T2	-0.0003	0.831	0.0019	0.379	<0.0001	0.911	<0.0001	0.982	-0.0006	0.098	-0.0010	0.088

NEU-T2	-0.0005	0.791	-0.0022	0.401	-0.0001	0.773	-0.0001	0.843	0.0006	0.192	0.0012	0.093
GWG/week	-0.0055	0.265	0.0027	0.683	-0.0005	0.565	0.0005	0.654	-0.0003	0.811	-0.0009	0.632
CRP	-		0.0001	0.060	-		<0.0001	0.810	-		<0.0001	0.868
GWG	-		-0.0004	0.153	-		<0.0001	0.381	-		<0.0001	0.758
Birth weight	-		-0.0001	0.165	-		<0.0001	0.290	-		<0.0001	0.809
Height	-		0.0115	0.158	-		0.0016	0.258	-		0.0008	0.746
FPR	-		0.0044	0.924	-		-0.0067	0.392	-		0.0166	0.191
PI	-		0.0703	0.169	-		0.0092	0.289	-		0.0045	0.784

p-BMI, pre-pregnancy BMI; TG, total triglycerides; TCHO, total cholesterol; HDL, high-density lipoprotein; LDL, low-density lipoprotein; WBC, white blood cell; NEU, neutrophil; CRP, C-reactive protein; GWG, gestational weight gain; FPR, Fetal-placenta ratio; PI, Ponderal Index.

Table S4. Capacity for GDM determination using acetic, propionic and butyric acids alone and in combination.

Variables	AUC (95%CI)	Cut-off value	Sensitivity	Specificity	*P*-value
Acetic	0.623(0.471-0.774)	0.171	66.7%	59.0%	<0.001
Propionic	0.738(0.597-0.879)	0.005	88.9%	61.5%	0.002
Butyric	0.655(0.500-0.810)	0.005	66.7%	66.7%	<0.001
Acetic + Propionic	0.756(0.615-0.898)	Pre=0.287	83.3%	66.7%	0.005
Acetic + Butyric	0.711(0.577-0.845)	Pre=0.247	94.4%	56.4%	<0.001
Propionic + Butyric	0.745(0.605-0.885)	Pre=0.290	83.3%	64.1%	0.003
Acetic + Propionic + Butyric	0.776(0.647-0.906)	Pre=0.299	83.3%	66.7%	0.007
Acetic + Propionic + Butyric+WBC-T2	0.823(0.698-0.949)	Pre=0.368	83.3%	79.5%	0.031
